# Harnessing the PI3K/Akt/mTOR pathway in T-cell acute lymphoblastic leukemia: Eliminating activity by targeting at different levels

**DOI:** 10.18632/oncotarget.579

**Published:** 2012-08-09

**Authors:** Daniela Bressanin, Camilla Evangelisti, Francesca Ricci, Giovanna Tabellini, Francesca Chiarini, Pier Luigi Tazzari, Fraia Melchionda, Francesca Buontempo, Pasqualepaolo Pagliaro, Andrea Pession, James A. McCubrey, Alberto M. Martelli

**Affiliations:** ^1^ Department of Human Anatomy, University of Bologna, Bologna, Italy; ^2^ Institute of Molecular Genetics, National Research Council-Rizzoli Orthopedic Institute, Bologna, Italy; ^3^ Immunohaematology and Transfusion Center, Policlinico S.Orsola-Malpighi, Bologna, Italy; ^4^ Department of Biomedical Sciences and Biotechnology, University of Brescia, Brescia, Italy; ^5^ Paediatric Oncology and Hematology Unit Lalla Seràgnoli, University of Bologna, Bologna, Italy; ^6^ Department of Microbiology & Immunology, School of Medicine, East Carolina University, Greenville, NC, USA

**Keywords:** acute leukemia, targeted therapy, signal transduction modulators, PI3K/PDK1, vertical inhibition

## Abstract

T-cell acute lymphoblastic leukemia (T-ALL) is an aggressive malignant hematological disorder arising in the thymus from T-cell progenitors. T-ALL mainly affects children and young adults, and remains fatal in 20% of adolescents and 50% of adults, despite progress in polychemotherapy protocols. Therefore, innovative targeted therapies are desperately needed for patients with a dismal prognosis. Aberrant activation of PI3K/Akt/mTOR signaling is a common event in T-ALL patients and portends a poor prognosis. Preclinical studies have highlighted that modulators of PI3K/Akt/mTOR signaling could have a therapeutic relevance in T-ALL. However, the best strategy for inhibiting this highly complex signal transduction pathway is still unclear, as the pharmaceutical companies have disclosed an impressive array of small molecules targeting this signaling network at different levels. Here, we demonstrate that a dual PI3K/PDK1 inhibitor, NVP-BAG956, displayed the most powerful cytotoxic effects against T-ALL cell lines and primary patients samples, when compared with a pan class I PI3K inhibitor (GDC-0941), an allosteric Akt inhibitor (MK-2206), an mTORC1 allosteric inhibitor (RAD-001), or an ATP-competitive mTORC1/mTORC2 inhibitor (KU-63794). Moreover, we also document that combinations of some of the aforementioned drugs strongly synergized against T-ALL cells at concentrations well below their respective IC_50_. This observation indicates that vertical inhibition at different levels of the PI3K/Akt/mTOR network could be considered as a future innovative strategy for treating T-ALL patients.

## INTRODUCTION

T-cell acute lymphoblastic leukemia (T-ALL) is a group of neoplastic disorders, arising in the thymus, that affect lymphoblasts committed to the T-cell lineage [1, 2]. T-ALL represents approximately 15% and 25% of pediatric and adult ALL cases, respectively, and mortality from T-ALL is still 20% for children and about 40-50% for adults [2]. For this reason, many research efforts are currently devoted to the development of targeted therapies to limit side effects of chemotherapy and to increase treatment efficacy for poor prognosis patients [3]. T-ALL blast cells display different molecular characteristics that affect disease evolution and prognosis [4]. Recently, it has been demonstrated that the same histopathological phenotype could be initiated by different point mutations, translocations, amplifications, deletions, and epigenetic modulation of gene expression that may contribute to the development of the cancer [5]. Actually, in different and potentially oncogenic strategic points, mutations arise and allow the tumor cells to support their proliferation and survival [6]. The PI3K/Akt/mTOR cascade is a crucial signal transduction pathway involved in cell growth, survival, and drug-resistance [7, 8]. Cancer cells, that escape the physiological regulation of this axis, increase their survival and proliferation [9]. Therefore, it is of great importance to study new therapeutic strategies to inhibit this signaling pathway. PI3K/Akt/mTOR constitutive activation is linked both to the pathogenesis and to progression of a wide variety of human cancers, including T-ALL [10-12]. In 50-75% of T-ALL patients, this pathway is constitutively active and negatively affects patient outcome [13]. Although several preclinical studies indicated that inhibition of PI3K/Akt/mTOR signaling could be an effective treatment for targeted therapy of T-ALL [13], it is still unclear which is the best target in this highly complex and branched signaling network. Indeed, pharmaceutical companies have disclosed an impressive array of inhibitors, targeting various components of this cascade [14, 15].

With the above in mind, we decided to undertake a comprehensive study where different inhibitors were tested under the same conditions, against T-ALL cells (both cell lines and primary samples from patients) displaying constitutive PI3K/Akt/mTOR activation. We analyzed the cytotoxic effects of a pan class I PI3K inhibitor (GDC-0941, [16]), an allosteric Akt inhibitor (MK-2206 [17]), a dual PI3K/PDK1 inhibitor (NVP-BAG956, [18]), an allosteric mTOR inhibitor (RAD-001 or Everolimus [19]), and an mTOR complex 1 (mTORC1)/mTOR complex 2 (mTORC2) ATP-competitive inhibitor (KU-63794 [20]). Some of the compounds we tested, have been approved (RAD-001) or have entered phase I/II clinical trials (GDC-0941, MK-2206) for solid tumor treatment. Here, we demonstrated that some of these drugs had a strong cytotoxic activity against T-ALL cell lines and primary cells. NVP-BAG956 displayed the highest efficacy. The combined use of some of these compounds was highly synergistic. We also documented the cytotoxic effects of NVP-BAG956 and MK-2006 against a T-ALL cell subpopulation (CD34^+^/CD7,^−^/CD4^−^) enriched for cancer stem cells (or leukemia initiating cells, LICs). The use of compounds able to eradicate LICs could reduce the percentage of treatment failures and decrease the relapse risk of T-ALL patients.

## RESULTS

### Inhibitors of PI3K/Akt/mTOR signaling are cytotoxic to T-ALL cell lines

The effects of inhibitors of PI3K/Akt/mTOR signaling on T-ALL cells were first analyzed by treating the cells with increasing concentrations of the drugs for 24 h and then evaluating the rates of survival by MTT assays. It is worth recalling here that all the T-ALL cell lines we used are PTEN-negative and display a defective p53 pathway [21]. Moreover, Jurkat cells do not express the inositol 5-phosphatase SHIP1 [22]. Both PTEN and SHIP1 are negative regulators of PI3K/Akt/mTOR signaling [23].

**Figure 1:**
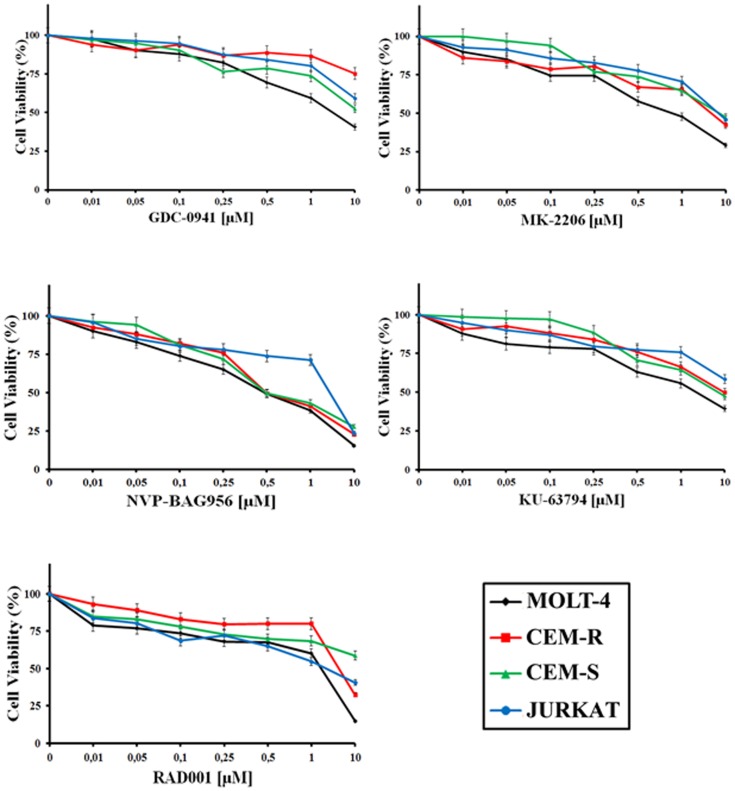
Inhibitors of PI3K/Akt/mTOR signaling affect viability of T-ALL cell lines MTT assays on T-ALL cell lines treated with GDC-0941, MK-2206, NVP-BAG956, KU-63794, RAD-001 for 24 h. The results are the mean of three different experiments ± s.d.

GDC-0941, a pan class I PI3K inhibitor, was effective on MOLT-4 cells (IC_50_= 2.8 μM), whereas CEM-S, and Jurkat cells displayed a much lower sensitivity (IC_50_ > 8.2 μM) (Table 1 and Fig. 1). CEM-R cells, that overexpress the ABCB1 drug transporter (also referred to as 170-KDa P-glycoprotein, one of major determinants of drug-resistance [24]), were resistant to GDC-0941. MK-2206 (an allosteric Akt inhibitor) was effective in both CEM-S and MOLT-4 cells (IC_50_ around 1.3-1.4 μM) whereas its cytotoxic effects on CEM-R and Jurkat cells were much lower (Table 1 and Fig. 1). Overall, NVP-BAG956, a dual PI3K/PDK1 inhibitor, was more effective than any other inhibitors tested. Most cell lines displayed an IC_50_ for NVP-BAG956 near to or lower than 1 μM, with the MOLT-4 cell line having the highest sensitivity to the drug (IC_50_= 500 nM) (Table 1 and Fig. 1). The allosteric mTORC1 inhibitor, RAD-001, was maximally efficacious on MOLT-4 (IC_50_= 900 nM), while Jurkat and CEM-R cells were less sensitive. The IC_50_ for RAD-001 on CEM-S cells was not achieved within the concentration range we utilized (Table 1 and Fig. 1). Finally, KU-63794, a dual ATP-competitive mTORC1/mTORC2 inhibitor, was effective on MOLT-4 and CEM-S cells (IC_50_= 3-4 μM), while Jurkat and CEM-R cells displayed a much higher IC_50_ (Table 1 and Fig. 1).

**Table 1:** IC_50s_ [μM] of PI3K/Akt/mTOR inhibitors tested against T-ALL cells lines T-ALL cells were treated with the drugs for 24 h, then the rates of survival were evaluated by MTT assays. n.a.: IC_50_ not achieved within the concentration range utilized during the present study (0.1-20 μM).

	COMPOUND	GDC-0941	MK-2206	NVP-BAG956	KU-63794	RAD-001
T-ALL cell lines	CEM-R	n.a.	6.20	0.95	10.10	5.80
	CEM-S	8.20	1.40	1.14	4.20	n.a.
	JURKAT	15.00	8.80	2.14	17.30	3.04
	MOLT-4	2.80	1.30	0.54	3.00	0.90

### Inhibitors of PI3K/Akt/mTOR signaling block cells in the G_0_/G1 phase of the cell cycle and induce apoptosis

**Figure 2:**
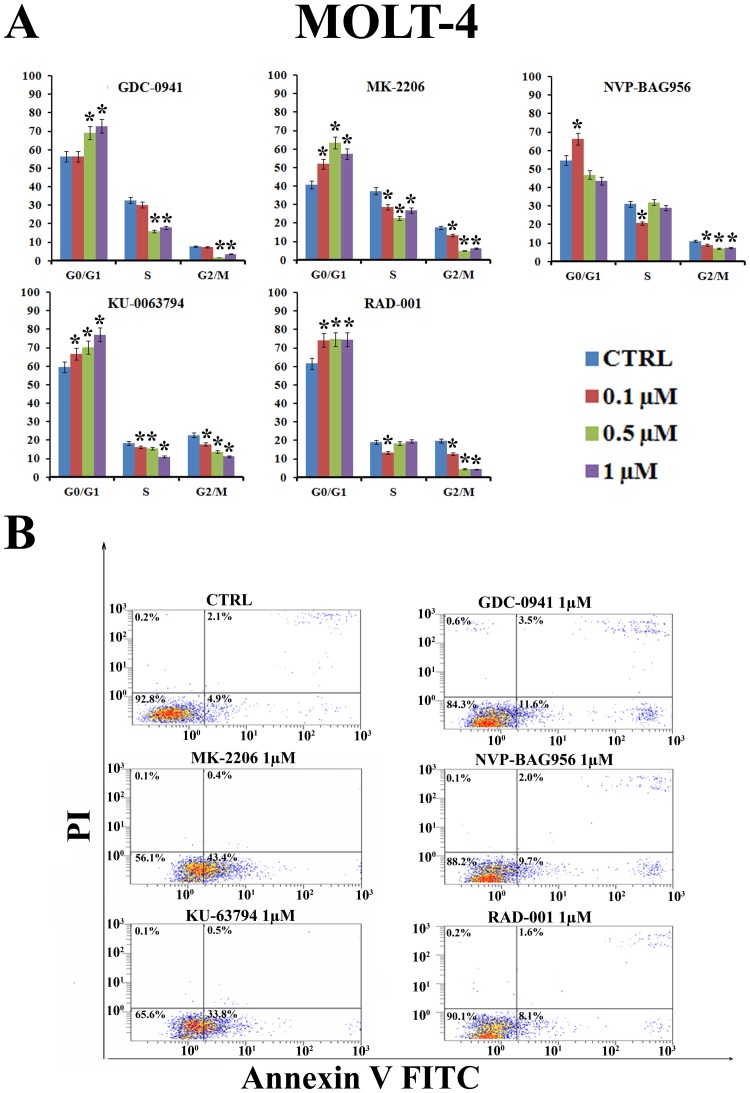
PI3K/Akt/mTOR signaling modulators affect cycle progression and induce apoptosis in MOLT-4 cells (A) MOLT-4 cell line was treated for 24 h with increasing concentrations of GDC-0941, MK-2206, NVP-BAG956, RAD-001 and KU-63794. Then, cell cycle analysis was performed by flow cytometry. One representative of three separate experiments ± s.d. is shown that yielded similar results. CTRL: control cells. Asterisks indicate significant differences (p <0.05) relative to control cells. (B) MOLT-4 cell line was treated for 24 h with GDC-0941, MK-2206, NVP-BAG956, RAD-001 and KU-63794 (1 μM). Then, cells were stained with Annexin V-FITC/PI and analyzed by flow cytometry. One representative of three separate experiments is shown that yielded similar results. CTRL: control cells.

To determine whether treatment of T-ALL cell lines with inhibitors of PI3K/Akt/mTOR signaling could affect cell cycle progression, MOLT-4 cells were incubated for 24 h with increasing concentrations of the drugs (0.1-1 μM) and the cell cycle was studied by means of flow cytometric analysis of propidium iodide (PI) -stained samples. All the drugs induced a statistically significant G_0_/G1 block and a concomitant decrease in both S and G_2_/M phases of the cell cycle (Fig. 2A). The induction of apoptosis was investigated by means of Annexin V-FITC/PI staining and flow cytometric analysis in MOLT-4 cells. The drugs that most potently induced apoptosis were MK-2206 and KU-63794 (Fig. 2B).

### Effects of the inhibitors on PI3K/Akt/mTOR signaling in T-ALL cell lines

**Figure 3:**
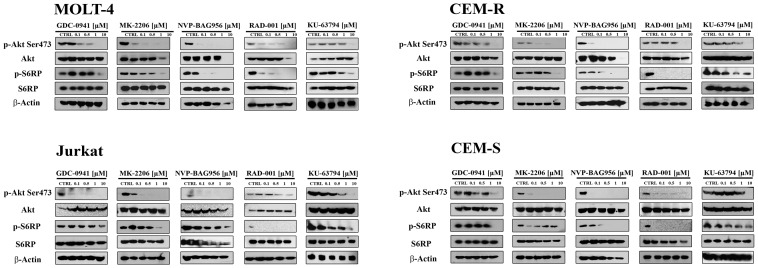
Effect of the signal transduction modulators on the phosphorylation status of PI3K/Akt/mTOR signaling components MOLT-4, Jurkat, CEM-R, and CEM-S cell lines were treated with increasing concentrations of GDC-0941, MK-2206, NVP-BAG956, RAD-001, and KU-63794 for 24 h. Then, cells were collected, lysed, and analyzed by western blotting. An antibody to β-actin documented equal lane loading (50 μg of protein/lane).

**Figure 4:**
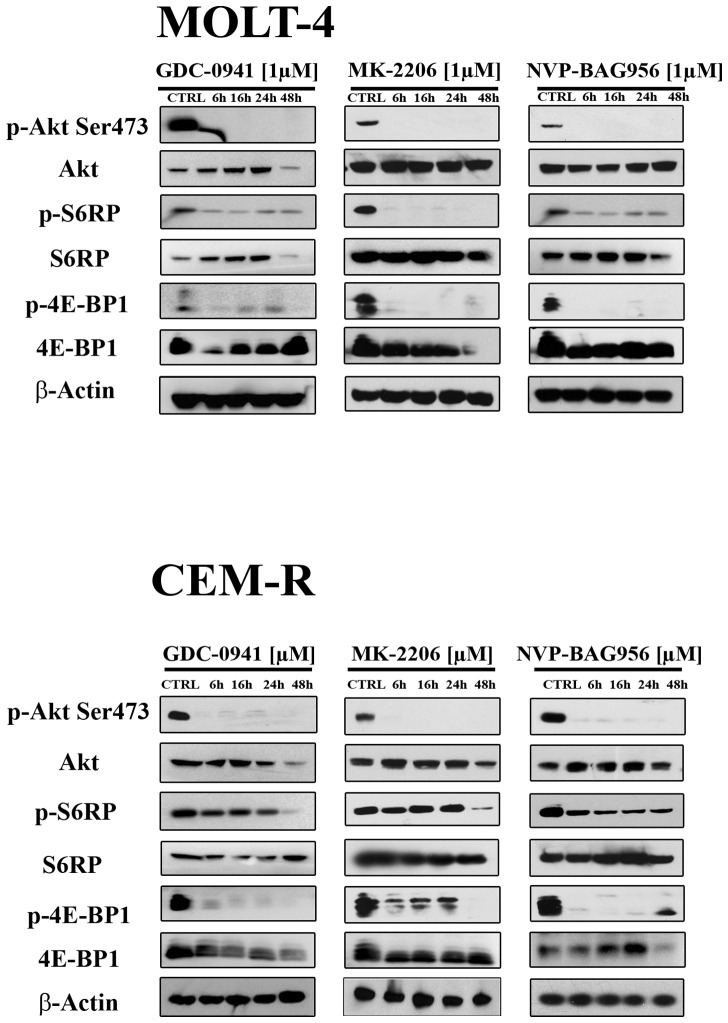
Time course of the phosphorylation status of PI3K/Akt/mTOR signaling components in response to drug treatment MOLT-4 (A) and CEM-R (B) cell lines were treated with 1 μM of GDC-0941, MK-2206, or NVP-BAG956, for 6, 16, 24 and 48 h, then they were collected, lysed, and analyzed by western blot. An antibody to β-actin documented equal lane loading (50 μg of protein/lane).

Western blot analysis demonstrated a concentration-dependent decrease in Ser 473 p-Akt, indicative of mTORC2 inhibition [25], after 24 h of treatment with all the PI3K/Akt/mTOR inhibitors, in all the cell lines analyzed (Fig. 3). Total Akt levels were unaffected by the drugs, except for NVP-BAG956 at the highest concentration employed. S6 ribosomal protein (S6RP), an mTORC1 downstream substrate [26], was also efficiently dephosphorylated by the inhibitors (Fig. 3). A time-dependent study was also performed and documented that, in MOLT-4 and in CEM-R cell lines, GDC-0941, MK-2206, and NVP-BAG956 (1 μM) dephosphorylated Ser 473 p-Akt, p-S6RP, and p-4E-BP1 (another mTORC1 downstream target [27]) already after 6 h of treatment (Fig. 4).

### Inhibitors of PI3K/Akt/mTOR signaling synergize together

**Figure 5:**
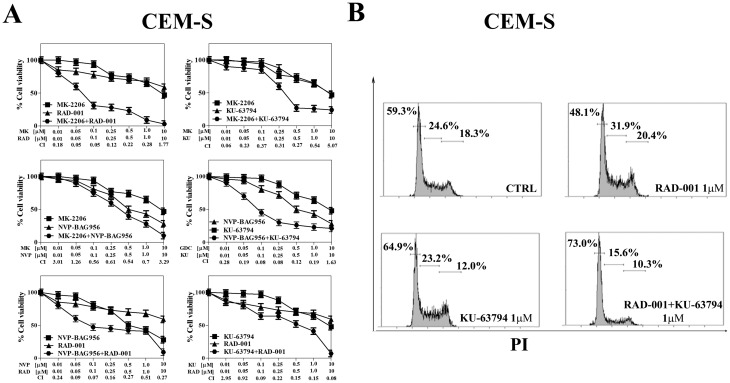
Vertical inhibition of the PI3K/Akt/mTOR pathway is synergistic in CEM-S cells (A) CEM-S cell lines were treated for 24 h with either drug alone or the combination of the two drugs at a fixed ratio. The combination index (CI) value for each data point was calculated with the appropriate software for dose effect analysis (CalcuSyn). Results are the mean of three different experiments ± s.d. (B) CEM-S cell line was treated for 24 h either RAD-001or KU-63794 alone, and with a combination of the two drugs at the same concentration (1 μM). Then, cell cycle analysis was performed by flow cytometry. One representative of three separate experiments is shown that yielded similar results. CTRL: control cells.

Then, it was investigated whether GDC-0941, MK-2206, NVP-BAG956, KU-63794, and RAD-001 could mutually synergize in T-ALL cells. CEM-S cells were incubated for 24 h with either one drug alone or with a combination of two drugs at an equal ratio. MTT assays were then performed. The less effective combinations were those consisting of GDC-0941/KU-63794, GDC-0941/MK-2206, GDC-0941/NVP-BAG965, GDC0941/RAD-001, MK-2206/NVP-BAG965. Indeed, with these combined treatments, an antagonism was frequently detected, and, when a synergism was observed, the combination index (CI) was usually not lower than 0.6, indicating a weak synergism (data not shown and Fig. 5A).

In contrast, a strong synergism (CI <0.3) was observed with MK-2206/RAD-001, MK-2206/KU-63794, NVP-BAG956/KU-63794, NVP-BAG956/RAD-001, and RAD-001/KU-63794 combinations (Fig. 5A). Notably, result analysis documented the existence of strong synergisms at drug concentrations well below the respective IC_50_ for these drugs in CEM-S cells.

Furthermore, we analyzed the effects of the RAD-001/KU-63794 combination on cell cycle progression, as these two drugs strongly synergized at 1 μM (CI<0.15). It is worth emphasizing here that in CEM-S cells the IC_50_ for KU-63794 was 4.2 μM, whereas the IC_50_ for RAD-001 was not achieved (see Table 1). After 24 h of administration of the drug combination, it was clearly noticeable a marked increase in the percentage of G_0_/G1 cells and a concomitant decrease in S and G_2_/M cells when compared with treatment with either drug alone (Fig. 5B).

### Inhibitors of PI3K/Akt/mTOR signaling have cytotoxic effects on T-ALL patient samples

**Figure 6:**
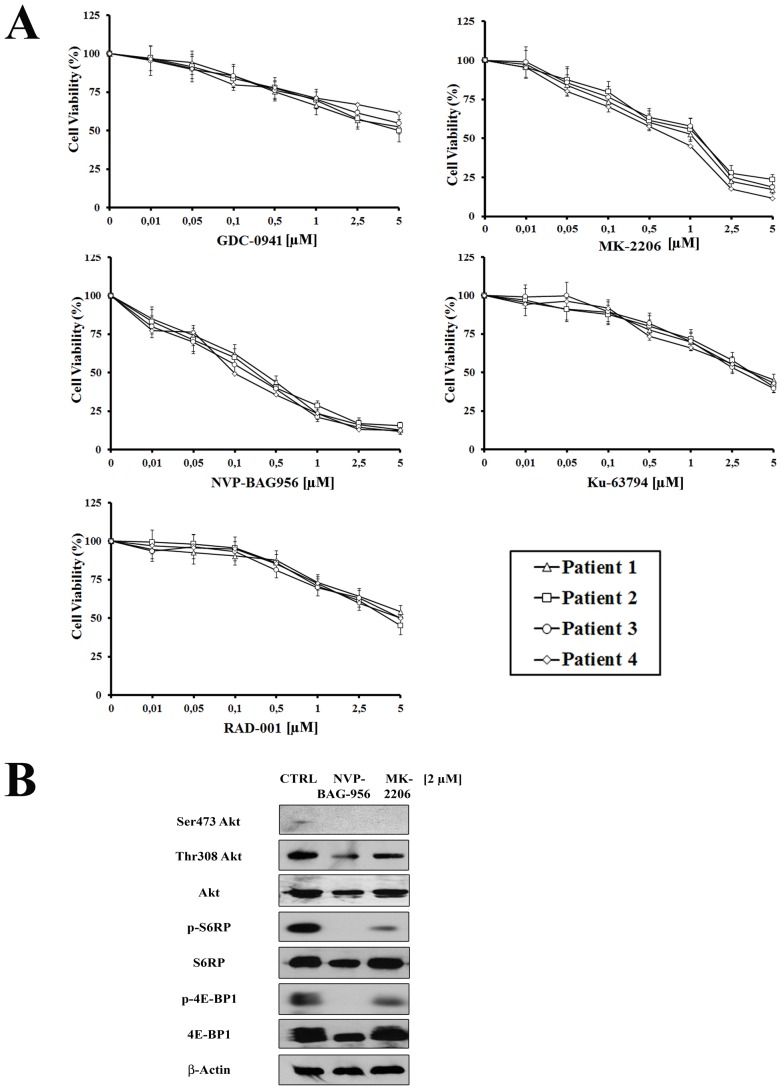
Inhibitors of PI3K/Akt/mTOR signaling are cytotoxic to T-ALL primary cells (A) MTT assay on four representative patient samples treated for 96 h with 10 ng/ml interleukin-7 and increasing concentrations of GDC-0941, MK-2206, NVP-BAG956, KU-63794, or RAD-001. Results are the mean of at three different experiments ± s.d. (B) Western blot analysis of a representative patient samples treated for 48 h with 2 μM MK-2206 or NVP-BAG956. Fifty μg of each lysate were electrophoresed on SDS-PAGE, transferred to a nitrocellulose membrane and probed with the appropriate antibodies. One representative of two different experiments is shown. CTRL: control cells.

To better evaluate the effectiveness of PI3K/Akt/mTOR inhbitors as potential therapeutic agents in T-ALL, we examined 6 pediatric T-ALL patient samples, isolated from bone marrow or peripheral blood and characterized by constitutive activation of the pathway. The effects of PI3K/Akt/mTOR signaling inhibitors on T-ALL lymphoblast samples, grown in the presence of interleukin-7 (IL-7), were evaluated by first treating the cells with increasing concentrations of the drugs and then analyzing the rates of survival by MTT assays. Four representative patients are presented in Fig. 6A. A marked reduction of cell viability at 96 h was detected. The two most powerful drugs were NVP-BAG956 (IC_50_ about 0.14 μM) and MK-2206 (IC_50_ about 0.44 μM).

For this reason, we performed western blot analysis on patient samples treated for 48 h with MK-2206 and NVP-BAG956, which demonstrated a decrease in the levels of Thr 308 p-Akt, Ser 473 p-Akt, p-4E-BP1, and p-S6RP, while their total levels of expression did not change.

### PI3K/Akt/mTOR signaling inhibitors activate caspase-3 and induce apoptosis in T-ALL lymphoblasts

**Figure 7:**
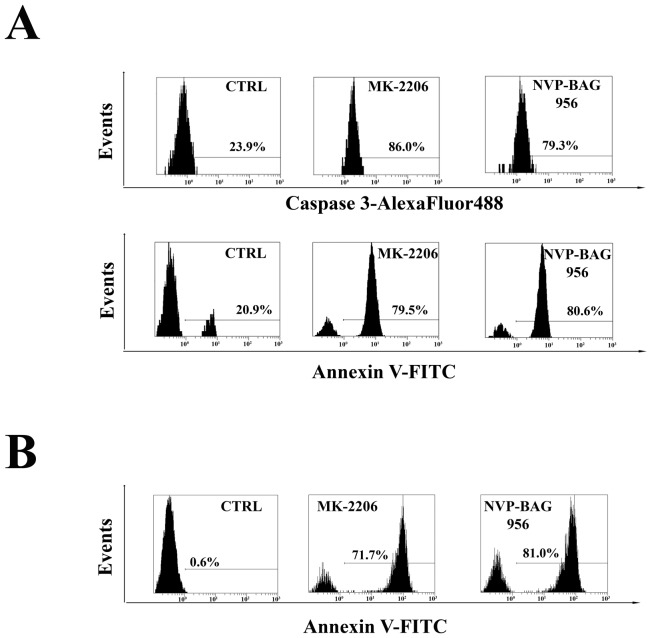
ModeInhibitors of PI3K/Akt/mTOR signaling induce apoptosis in T-ALL lymphoblasts and putative LICs (A) Flow cytometric analysis of patient samples treated for 24 h with 2 μM MK-2206 or NVP-BAG956. Cells were stained with AlexaFluor® 488-conjugated anti-cleaved caspase-3 or with Annexin V-FITC/PI. The percentages of positive cells are indicated. The plots are representative of three separate experiments which were performed in duplicate. (B) Flow cytometric analysis of patient samples enriched for LICs, treated for 48 h with 2 μM MK-2206 or NVP-BAG956. The CD34^+^/CD7^−^/CD4^−^ subset was stained with Annexin-FITC, to analyze apoptosis induction by the drugs (2 μM). In A and B one representative of two different experiments is shown. CTRL: control cells.

T-ALL lymphoblasts samples were analyzed to evaluate the levels of cleaved caspase-3 and the induction of apoptosis in response to treatment with MK-2206 or NVP-BAG956. Flow cytometric analysis documented that the drugs caused an increase in cleaved caspase-3 and an induction of apoptosis, as documented by Annexin V-FITC/PI staining (Fig. 7A).

### Inhibitors of PI3K/Akt/mTOR signaling induce apoptosis in the CD34^+^/CD7^−^/CD4^−^ subset of patient lymphoblasts

Finally, using quadruple staining and flow cytometric analysis, we investigated whether MK-2206 and NVP-BAG956 could induce apoptosis in a T-ALL patient lymphoblast subset (CD34^+^/CD7^−^/CD4^−^), which is enriched in putative LICs [28]. After electronic gating on the CD7^−^/CD4^−^ lymphoblast subset, cells were analyzed for CD34 expression and positivity to Annexin V staining. After 48 h of treatment, the drugs markedly induced apoptosis in the CD34^+^/CD7^−^/CD4^−^ subpopulation (Fig. 7B). NVP-BAG956 was slightly more powerful than MK-2206, even when used at an equimolar concentration.

## DISCUSSION

PI3K/Akt/mTOR signaling dysregulation play a key role in the onset of human cancers [29, 30]. Indeed, constitutive activation of this axis is associated with aberrant cell survival [15] and controls neoplastic motility, invasion, and metastasis [31]. Recent studies have suggested that this axis could be a promising target in T-ALL [32, 33], as in more than 70% of T-ALL patients, PI3K/Akt/mTOR signaling is constitutively activated and portends a poor prognosis [34]. In light of this, it is very important to develop new therapeutic strategies against T-ALL cells aimed to negatively modulate this signal cascade for improving the clinical outcome of the patients.

Since aberrant PI3K/Akt/mTOR pathway activation plays a crucial role in the pathogenesis of T-ALL, the aim of this research has been to test and compare the therapeutic potential of selective inhibitors, such as GDC-0941, MK-2206, NVP-BAG956, RAD-001, and KU-63794. In this study, we tested these drugs either alone or in combination, against T-ALL cell lines and primary samples from T-ALL patients. The highest cytotoxic potential against T-ALL cell lines and patient lymphoblasts was displayed by NVP-BAG956, a dual PI3K/PDK1 inhibitor which has been shown to be effective against BCR-ABL- and mutant FLT3-expressing acute leukemia cells [18]. Subsequently, NVP-BAG956 has been documented to affect proliferation of melanoma cells [35]. To our knowledge this is the first time this drug is used against T-ALL cells. NVP-BAG956 was mainly cytostatic in T-ALL cell lines and was not a strong inducer of apoptosis. However, it potently induced apoptosis in T-ALL primary cells, including a cell subset that is enriched in putative LICs. GDC-0941 is an inhibitor of class I PI3K that has entered clinical trials for solid tumors [16]. In T-ALL cell lines and patient samples, GDC-0941 displayed a weak cytostatic effect. MOLT-4 cells were more sensitive to GDC-0941 than the other cell lines. The allosteric Akt inhibitor MK-2206 [36], that is presently undergoing clinical trials for the treatment of solid tumors [17], was more powerful than GDC-0941 in both T-ALL cell lines and primary samples. Apart from being cytostatic, MK-2206 also induced apoptosis.

Surprisingly, we found that RAD-001 (an allosteric mTORC1 inhibitor belonging to the rapalog class [37]) was more powerful than KU-63794, an ATP-competitive mTORC1/mTORC2 inhibitor [20], especially in MOLT-4 cells. Indeed, ATP-competitive mTORC1/mTORC2 inhibitors are generally considered to be more powerful than rapamycin and rapalogs [38]. However, RAD-001 and KU-63794 displayed nearly similar weak potency against T-ALL lymphoblasts.

An interesting observation is that RAD-001 treatment resulted in Ser 473 p-Akt dephosphorylation in T-ALL cell lines. In most cancer cell types, rapalogs such as RAD-001, increased Akt phosphorylation through inhibition of a negative feed-back loop based on mTORC1/p70S6K/IRS1/PI3K [39]. Inhibition of such a negative feed-back loop up-regulates mTORC2-dependent phosphorylation of Akt on Ser 473 and increases cell survival [40]. However, the rapalog inhibitor CCI-779 has been reported to cause mTORC2 disassembly and Ser 473 p-Akt dephosphorylation [41]. Thus, it may be that RAD-001 disassembled mTORC2 complex in T-ALL cell lines. This finding seems also to indicate that rapamycin and RAD-001 effects are not superimposable, as rapamycin treatment of T-ALL cell lines, under the same conditions employed here as for RAD-001, did not result in Ser 473 p-Akt dephosphorylation in the same T-ALL cell lines [21].

A rapidly emerging theme in targeted therapy of PI3K/Akt/mTOR signaling, is that combined “vertical” inhibition at different nodes of the cascade often leads to better results that the use of either single or dual inhibitors. However, most of the studies performed in this field so far took advantage of solid tumor models [42-44]. As far as we know, this is the first report which documented the superior efficacy of vertical targeting of the PI3K/Akt/mTOR pathway in T-ALL cell lines. Previous evidence has demonstrated that the PI3K/Akt/mTOR network is characterized by multiple feed-back loops that finely act to regulate signal transduction [45]. Hence, the existence of these loops could limit the antitumor effects of PI3K/Akt/mTOR inhibitors given in monotherapy settings, and explains the importance of testing the effects of combination treatment. Consequently, inhibiting at the same time at different levels and with different inhibitors the PI3K/Akt/mTOR pathway is a possible strategy to enhance their effectiveness on leukemic cells. It is remarkable that in T-ALL cell lines, a synergism was detected for drugs used at various concentrations that were considerably below the IC_50_ of the drugs when administered alone. The most effective drug combinations in T-ALL lines were those consisting of MK-2206/RAD-001, MK-2206/KU-63794, NVP-BAG956/KU-63794, NVP-BAG956/RAD-001, and RAD-001/KU-63794. These findings could have a clinical relevance for T-ALL patients. Indeed, as combinations of these drugs increased the cytotoxicity, the use of a much lower concentration of the inhibitors was possible and could considerably attenuate the toxic side effects. Experiments are underway to better understand the molecular mechanisms underlying the increased cytotoxic effects of these combinations.

Moreover, it is important to emphasize that, in T-ALL patients lymphoblasts, both MK-2206 and NVP-BAG956 were cytotoxic to putative LICs. LICs express surface markers normally exhibited by stem cells and they are more resistant to various chemotherapies [46]. Strategies that eliminate these cells could have significant clinical implications [47, 48]. In conclusion, our results demonstrated that targeting PI3K/Akt/mTOR pathway at different levels in T-ALL cell lines resulted in an increase of cytotoxic effects and then at least some of tested inhibitors may represent promising drugs also for their capacity to target T-ALL LICs.

## MATERIALS AND METHODS

### Materials

GDC-0941 and NVP-BAG956 were purchased from Axon Medchem BV (Groningen, The Netherlands), while MK-2206, KU-63794, and RAD-001 were purchased from Selleck Chemicals (Houston, TX, USA). For western blotting, primary antibodies were bought from Cell Signaling Technology (Danvers, MA, USA). For flow cytometric analysis, AlexaFluor ® 488-conjugated antibody to cleaved caspase-3 was from Beckman Coulter (Miami, FL, USA).

### Cell culture and primary samples

The T-ALL cell lines Jurkat, MOLT-4, CEM-S, and CEM-R (CEM VBL100, drug-resistant cells overexpressing the ABCB1 drug transporter [49]) were grown in RPMI 1640, supplemented with 10% fetal bovine serum (FBS), L-glutamine, and penicillin-streptomycin (Sigma Aldrich, St Louis, MO, USA). Samples from T-ALL pediatric patients were obtained with informed consent according to institutional guidelines and isolated using Ficoll-Paque (Amersham Biosciences, Little Chalfont, UK) [50, 51] and were grown in complete medium [RPMI 1640 supplemented with 20% FBS, ITS (insulin-transferrin-sodium selenite), and amphotericin B].

### Cell viability analysis

MTT (3-[4,5-Dimethylthythiazol-2-yl]-2,5-Diphenyltetrazolium Bromide) assays were performed to assess the sensitivity of cells to drugs, as previously described [52]. In particular, T-ALL patient lymphoblasts (2 × 10^6^ cells/ml) were cultured in triplicate in flat-bottomed 96-well plates at 37°C with 5% CO_2_. Cultures were carried out for 96 h in complete medium supplemented with 10 ng/ml IL-7. Results were statistically analyzed by GraphPadPrism Software (GraphPad Software Inc., San Diego, CA, USA).

### Cell cycle analysis

Flow cytometric analysis was performed using a PI/RNase A staining according to standard procedures, as described previously [53, 54]. Samples were analyzed on a FC500 flow cytometer (Beckman Coulter) with the appropriate software (CXP, Beckman Coulter).

### Flow cytometric analysis of Annexin V-FITC in T-ALL cell lines and patient samples

After in vitro drug treatment, T-ALL cell lines and patient lymphoblasts were washed twice in PBS, labeled with Annexin V-FITC in binding buffer, stained with PI, and then analyzed by flow cytometry on an FC500 flow cytometer [55].

### Western blot analysis

This was performed by standard methods, as previously reported [56]. Analysis with an antibody to β-actin demonstrated equal protein loading.

### Combined drug effect analysis

The combination effect and potential synergy were evaluated from quantitative analysis of dose-effect relationships, as described previously [57]. For each combination experiment, a CI number was calculated using the CalcuSyn software (Biosoft, Cambridge, UK). This method of analysis generally defines CI values of 0.9 to 1.1 as additive, 0.3 to 0.9 as synergistic, and <0.3 as strongly synergistic, whereas values >1.1 are considered antagonistic.

### Flow cytometric analysis of cleaved caspase-3 levels in T-ALL patient samples

After in vitro treatment, T-ALL lymphoblasts were fixed in Reagent 1 of the Intraprep Kit (Beckman Coulter) and permeabilized with saponin-based Reagent 2, as reported elsewhere [49]. Cells were incubated with an anti-cleaved caspase-3 primary antibody conjugated to AlexaFluor® 488. A rabbit IgG conjugated to AlexaFluor® 488 was used as an irrelevant antibody. Cells were analyzed on a FC500 flow cytometer [49].

### Flow cytometric analysis of putative T-ALL LIC

This was performed essentially as previously reported [49]. To detect apoptotic cells, samples were incubated with Annexin V-FITC. Samples were analyzed on a Navios flow cytometer (Beckman Coulter) equipped with Kaluza software (Beckman Coulter).

### Statistical evaluation

The data are presented as mean values from three separate experiments ± s.d. Data were statistically analyzed by a Dunnet test after one-way analysis of variance (ANOVA) at a level of significance of p < 0.05 vs. control samples.

## References

[1] Demarest RM, Ratti F, Capobianco AJ (2008). It's T-ALL about Notch. Oncogene.

[2] Pui CH, Robison LL, Look AT (2008). Acute lymphoblastic leukaemia. Lancet.

[3] Dancey JE, Bedard PL, Onetto N, Hudson TJ (2012). The genetic basis for cancer treatment decisions. Cell.

[4] Kraszewska MD, Dawidowska M, Szczepanski T, Witt M (2012). T-cell acute lymphoblastic leukaemia: Recent molecular biology findings. Br J Haematol.

[5] Kelloff GJ, Sigman CC (2012). Cancer biomarkers: Selecting the right drug for the right patient. Nat Rev Drug Discov.

[6] Cairns RA, Harris IS, Mak TW (2011). Regulation of cancer cell metabolism. Nat Rev Cancer.

[7] Martelli AM, Evangelisti C, Follo MY, Ramazzotti G, Fini M, Giardino R, Manzoli L, McCubrey JA, Cocco L (2011). Targeting the phosphatidylinositol 3-kinase/Akt/mammalian target of rapamycin signaling network in cancer stem cells. Curr Med Chem.

[8] Steelman LS, Chappell WH, Abrams SL, Kempf RC, Long J, Laidler P, Mijatovic S, Maksimovic-Ivanic D, Stivala F, Mazzarino MC, Donia M, Fagone P, Malaponte G, Nicoletti F, Libra M, Milella M (2011). Roles of the RAF/MEK/ERK and PI3K/PTEN/AKT/mTOR pathways in controlling growth and sensitivity to therapy-implications for cancer and aging. Aging.

[9] Janes MR, Fruman DA (2010). Targeting TOR dependence in cancer. Oncotarget.

[10] Schmidt-Kittler O, Zhu J, Yang J, Liu G, Hendricks W, Lengauer C, Gabelli SB, Kinzler KW, Vogelstein B, Huso DL, Zhou S (2010). PI3Kα inhibitors that inhibit metastasis. Oncotarget.

[11] Peng C, Chen Y, Li D, Li S (2010). Role of PTEN in leukemia stem cells. Oncotarget.

[12] Kharas MG, Okabe R, Ganis JJ, Gozo M, Khandan T, Paktinat M, Gilliland DG, Gritsman K (2010). Constitutively active Akt depletes hematopoietic stem cells and induces leukemia in mice. Blood.

[13] Zhao WL (2010). Targeted therapy in T-cell malignancies: Dysregulation of the cellular signaling pathways. Leukemia.

[14] Markman B, Dienstmann R, Tabernero J (2010). Targeting the PI3K/Akt/mTOR pathway--beyond rapalogs. Oncotarget.

[15] Chappell WH, Steelman LS, Long JM, Kempf RC, Abrams SL, Franklin RA, Basecke J, Stivala F, Donia M, Fagone P, Malaponte G, Mazzarino MC, Nicoletti F, Libra M, Maksimovic-Ivanic D, Mijatovic S (2011). Ras/RAF/MEK/ERK and PI3K/PTEN/Akt/mTOR inhibitors: Rationale and importance to inhibiting these pathways in human health. Oncotarget.

[16] Raynaud FI, Eccles SA, Patel S, Alix S, Box G, Chuckowree I, Folkes A, Gowan S, De Haven Brandon A, Di Stefano F, Hayes A, Henley AT, Lensun L, Pergl-Wilson G, Robson A, Saghir N (2009). Biological properties of potent inhibitors of class I phosphatidylinositide 3-kinases: From PI-103 through PI-540, PI-620 to the oral agent GDC-0941. Mol Cancer Ther.

[17] Hirai H, Sootome H, Nakatsuru Y, Miyama K, Taguchi S, Tsujioka K, Ueno Y, Hatch H, Majumder PK, Pan BS, Kotani H (2010). MK-2206, an allosteric Akt inhibitor, enhances antitumor efficacy by standard chemotherapeutic agents or molecular targeted drugs in vitro and in vivo. Mol Cancer Ther.

[18] Weisberg E, Banerji L, Wright RD, Barrett R, Ray A, Moreno D, Catley L, Jiang J, Hall-Meyers E, Sauveur-Michel M, Stone R, Galinsky I, Fox E, Kung AL, Griffin JD (2008). Potentiation of antileukemic therapies by the dual PI3K/PDK-1 inhibitor, BAG956: Effects on BCR-ABL- and mutant FLT3-expressing cells. Blood.

[19] Populo H, Lopes JM, Soares P (2012). The mTOR signalling pathway in human cancer. Int J Mol Sci.

[20] Garcia-Martinez JM, Moran J, Clarke RG, Gray A, Cosulich SC, Chresta CM, Alessi DR (2009). Ku-0063794 is a specific inhibitor of the mammalian target of rapamycin (mTOR). Biochem J.

[21] Chiarini F, Fala F, Tazzari PL, Ricci F, Astolfi A, Pession A, Pagliaro P, McCubrey JA, Martelli AM (2009). Dual inhibition of class Ia phosphatidylinositol 3-kinase and mammalian target of rapamycin as a new therapeutic option for T-cell acute lymphoblastic leukemia. Cancer Res.

[22] Horn S, Endl E, Fehse B, Weck MM, Mayr GW, Jucker M (2004). Restoration of SHIP activity in a human leukemia cell line downregulates constitutively activated phosphatidylinositol 3-kinase/Akt/GSK-3β signaling and leads to an increased transit time through the G1 phase of the cell cycle. Leukemia.

[23] Martelli AM, Evangelisti C, Chiarini F, McCubrey JA (2010). The phosphatidylinositol 3-kinase/Akt/mTOR signaling network as a therapeutic target in acute myelogenous leukemia patients. Oncotarget.

[24] Fu D, Arias IM (2012). Intracellular trafficking of p-glycoprotein. Int J Biochem Cell Biol.

[25] Oh WJ, Jacinto E (2011). mTOR complex 2 signaling and functions. Cell Cycle.

[26] Binda M, Bonfils G, Panchaud N, Peli-Gulli MP, De Virgilio C (2010). An egocentric view of TORC1 signaling. Cell Cycle.

[27] Yellen P, Saqcena M, Salloum D, Feng J, Preda A, Xu L, Rodrik-Outmezguine V, Foster DA (2011). High-dose rapamycin induces apoptosis in human cancer cells by dissociating mTOR complex 1 and suppressing phosphorylation of 4E-BP1. Cell Cycle.

[28] Cox CV, Martin HM, Kearns PR, Virgo P, Evely RS, Blair A (2007). Characterization of a progenitor cell population in childhood T-cell acute lymphoblastic leukemia. Blood.

[29] Sokolosky ML, Stadelman KM, Chappell WH, Abrams SL, Martelli AM, Stivala F, Libra M, Nicoletti F, Drobot LB, Franklin RA, Steelman LS, McCubrey JA (2011). Involvement of Akt-1 and mTOR in sensitivity of breast cancer to targeted therapy. Oncotarget.

[30] Altman JK, Sassano A, Platanias LC (2011). Targeting mTOR for the treatment of AML. New agents and new directions. Oncotarget.

[31] Zhou H, Huang S (2011). Role of mTOR signaling in tumor cell motility, invasion and metastasis. Curr Protein Pept Sci.

[32] Martelli AM, Chiarini F, Evangelisti C, Cappellini A, Buontempo F, Bressanin D, Fini M, McCubrey JA (2012). Two hits are better than one: Targeting both phosphatidylinositol 3-kinase and mammalian target of rapamycin as a therapeutic strategy for acute leukemia treatment. Oncotarget.

[33] Martelli AM, Evangelisti C, Chappell W, Abrams SL, Basecke J, Stivala F, Donia M, Fagone P, Nicoletti F, Libra M, Ruvolo V, Ruvolo P, Kempf CR, Steelman LS, McCubrey JA (2011). Targeting the translational apparatus to improve leukemia therapy: Roles of the PI3K/PTEN/Akt/mTOR pathway. Leukemia.

[34] Jotta PY, Ganazza MA, Silva A, Viana MB, da Silva MJ, Zambaldi LJ, Barata JT, Brandalise SR, Yunes JA (2010). Negative prognostic impact of PTEN mutation in pediatric T-cell acute lymphoblastic leukemia. Leukemia.

[35] Marone R, Erhart D, Mertz AC, Bohnacker T, Schnell C, Cmiljanovic V, Stauffer F, Garcia-Echeverria C, Giese B, Maira SM, Wymann MP (2009). Targeting melanoma with dual phosphoinositide 3-kinase/mammalian target of rapamycin inhibitors. Mol Cancer Res.

[36] Tan S, Ng Y, James DE (2011). Next-generation Akt inhibitors provide greater specificity: Effects on glucose metabolism in adipocytes. Biochem J.

[37] Le Tourneau C, Faivre S, Serova M, Raymond E (2008). mTORC1 inhibitors: Is temsirolimus in renal cancer telling us how they really work?. Br J Cancer.

[38] Grzybowska-Izydorczyk O, Smolewski P (2012). mTOR kinase inhibitors as a treatment strategy in hematological malignancies. Future Med Chem.

[39] Tamburini J, Chapuis N, Bardet V, Park S, Sujobert P, Willems L, Ifrah N, Dreyfus F, Mayeux P, Lacombe C, Bouscary D (2008). Mammalian target of rapamycin (mTOR) inhibition activates phosphatidylinositol 3-kinase/Akt by up-regulating insulin-like growth factor-1 receptor signaling in acute myeloid leukemia: Rationale for therapeutic inhibition of both pathways. Blood.

[40] Meric-Bernstam F, Akcakanat A, Chen H, Do KA, Sangai T, Adkins F, Gonzalez-Angulo AM, Rashid A, Crosby K, Dong M, Phan AT, Wolff RA, Gupta S, Mills GB, Yao J (2012). PI3KCA/PTEN mutations and Akt activation as markers of sensitivity to allosteric mTOR inhibitors. Clin Cancer Res.

[41] Zeng Z, Sarbassov dos D, Samudio IJ, Yee KW, Munsell MF, Ellen Jackson C, Giles FJ, Sabatini DM, Andreeff M, Konopleva M (2007). Rapamycin derivatives reduce mTORC2 signaling and inhibit Akt activation in AML. Blood.

[42] Ren H, Chen M, Yue P, Tao H, Owonikoko TK, Ramalingam SS, Khuri FR, Sun SY (2012). The combination of RAD001 and NVP-BKM120 synergistically inhibits the growth of lung cancer in vitro and in vivo. Cancer Lett.

[43] Zito CR, Jilaveanu LB, Anagnostou V, Rimm D, Bepler G, Maira SM, Hackl W, Camp R, Kluger HM, Chao HH (2012). Multi-level targeting of the phosphatidylinositol-3-kinase pathway in non-small cell lung cancer cells. PloS One.

[44] Aziz SA, Jilaveanu LB, Zito C, Camp RL, Rimm DL, Conrad P, Kluger HM (2010). Vertical targeting of the phosphatidylinositol-3 kinase pathway as a strategy for treating melanoma. Clin Cancer Res.

[45] Dunlop EA, Tee AR (2009). Mammalian target of rapamycin complex 1: Signalling inputs, substrates and feedback mechanisms. Cell Signal.

[46] McCubrey JA, Steelman LS, Abrams SL, Misaghian N, Chappell WH, Basecke J, Nicoletti F, Libra M, Ligresti G, Stivala F, Maksimovic-Ivanic D, Mijatovic S, Montalto G, Cervello M, Laidler P, Bonati A (2012). Targeting the cancer initiating cell: The ultimate target for cancer therapy. Curr Pharm Des.

[47] Wang J, Liu Y, Tan LX, Lo JC, Du J, Ryu MJ, Ranheim EA, Zhang J (2011). Distinct requirements of hematopoietic stem cell activity and NRAS G12D signaling in different cell types during leukemogenesis. Cell Cycle.

[48] Kharas MG, Gritsman K (2010). Akt: A double-edged sword for hematopoietic stem cells. Cell Cycle.

[49] Evangelisti C, Ricci F, Tazzari P, Tabellini G, Battistelli M, Falcieri E, Chiarini F, Bortul R, Melchionda F, Pagliaro P, Pession A, McCubrey JA, Martelli AM (2011). Targeted inhibition of mTORC1 and mTORC2 by active-site mtor inhibitors has cytotoxic effects in T-cell acute lymphoblastic leukemia. Leukemia.

[50] Boehrer S, Galluzzi L, Lainey E, Bouteloup C, Tailler M, Harper F, Pierron G, Ades L, Thepot S, Sebert M, Gardin C, de Botton S, Fenaux P, Kroemer G (2011). Erlotinib antagonizes constitutive activation of src family kinases and mTOR in acute myeloid leukemia. Cell Cycle.

[51] Thepot S, Lainey E, Cluzeau T, Sebert M, Leroy C, Ades L, Tailler M, Galluzzi L, Baran-Marszak F, Roudot H, Eclache V, Gardin C, de Botton S, Auberger P, Fenaux P, Kroemer G (2011). Hypomethylating agents reactivate FOXO3a in acute myeloid leukemia. Cell Cycle.

[52] Lin YW, Beharry ZM, Hill EG, Song JH, Wang W, Xia Z, Zhang Z, Aplan PD, Aster JC, Smith CD, Kraft AS (2010). A small molecule inhibitor of PIM protein kinases blocks the growth of precursor T-cell lymphoblastic leukemia/lymphoma. Blood.

[53] Dufies M, Jacquel A, Robert G, Cluzeau T, Puissant A, Fenouille N, Legros L, Raynaud S, Cassuto JP, Luciano F, Auberger P (2011). Mechanism of action of the multikinase inhibitor foretinib. Cell Cycle.

[54] Evangelisti C, Gaboardi GC, Billi AM, Ognibene A, Goto K, Tazzari PL, McCubrey JA, Martelli AM (2010). Identification of a functional nuclear export sequence in diacylglycerol kinase-ζ. Cell Cycle.

[55] Jung AS, Kaushansky A, Macbeath G, Kaushansky K (2011). Tensin2 is a novel mediator in thrombopoietin (TPO)-induced cellular proliferation by promoting Akt signaling. Cell Cycle.

[56] Valenti F, Fausti F, Biagioni F, Shay T, Fontemaggi G, Domany E, Yaffe MB, Strano S, Blandino G, Di Agostino S (2011). Mutant p53 oncogenic functions are sustained by plk2 kinase through an autoregulatory feedback loop. Cell Cycle.

[57] Abrams SL, Steelman LS, Shelton JG, Chappell W, Basecke J, Stivala F, Donia M, Nicoletti F, Libra M, Martelli AM, McCubrey JA (2010). Enhancing therapeutic efficacy by targeting non-oncogene addicted cells with combinations of signal transduction inhibitors and chemotherapy. Cell Cycle.

